# Alterations in the human lung proteome with lipopolysaccharide

**DOI:** 10.1186/1471-2466-9-20

**Published:** 2009-05-11

**Authors:** Russell P Bowler, Nichole Reisdorph, Richard Reisdorph, Edward Abraham

**Affiliations:** 1 Department of Medicine, National Jewish Health, Denver, Colorado, USA; 2 Department of Medicine, University of Alabama at Birmingham, Birmingham, AL, USA

## Abstract

**Background:**

Recombinant human activated protein C (rhAPC) is associated with improved survival in high-risk patients with severe sepsis; however, the effects of both lipopolysaccharide (LPS) and rhAPC on the bronchoalveolar lavage fluid (BALF) proteome are unknown.

**Methods:**

Using differential in gel electrophoresis (DIGE) we identified changes in the BALF proteome from 10 healthy volunteers given intrapulmonary LPS in one lobe and saline in another lobe. Subjects were randomized to pretreatment with saline or rhAPC.

**Results:**

An average of 255 protein spots were detected in each proteome. We found 31 spots corresponding to 8 proteins that displayed abundance increased or decreased at least 2-fold after LPS. Proteins that decreased after LPS included surfactant protein A, immunoglobulin J chain, fibrinogen-γ, α_1_-antitrypsin, immunoglobulin, and α_2_-HS-glycoprotein. Haptoglobin increased after LPS-treatment. Treatment with rhAPC was associated with a larger relative decrease in immunoglobulin J chain, fibrinogen-γ, α_1_-antitrypsin, and α_2_-HS-glycoprotein.

**Conclusion:**

Intrapulmonary LPS was associated with specific protein changes suggesting that the lung response to LPS is more than just a loss of integrity in the alveolar epithelial barrier; however, pretreatment with rhAPC resulted in minor changes in relative BALF protein abundance consistent with its lack of affect in ALI and milder forms of sepsis.

## Background

Bacterial pneumonia and Gram negative sepsis (GNS) are syndromes of complex pathogenesis with a high mortality [1]. Although the pathophysiology of lung injury during sepsis is complex, the host response to lipopolysaccharide (LPS) is one of the early activators of the immune system [2]. Later characteristics of GNS and pneumonia include severe dysfunction of the alveolar-capillary barrier leading to acute lung injury (ALI) and respiratory failure [3]. In ALI, damage to the endothelium and alveolar epithelium barrier results in plasma protein efflux into the alveolar spaces that leads to hypoxemia and respiratory failure [4-8]. GNS and ALI are also characterized by systemic activation of the acute phase response with elevation of acute phase proteins in serum [9] and epithelial lining fluid [10].

Infection agents and inflammatory agents are also thought to induce coagulation by stimulating the expression of tissue factor on monocytes and endothelium, leading to thrombin generation [11]. Activated protein C (APC) is a natural anticoagulant that plays an important role in coagulation homeostasis by inactivating the procoagulant factors Va and VIIIa, resulting in decreased thrombin generation. rhAPC can improve lung function after ALI by improving PaO_2_/FIO_2 _ratios, reducing microvascular shunt fraction, decreasing peak airway pressures, and reducing lung nitrotyrosine formation, even without a significant anticoagulant effect [12]. Recombinant human APC (rhAPC) is the only medication that has been shown to reduce mortality in patients with severe sepsis [13]. Although rhAPC is inactivates clotting factors Va and VIIIa, there is increasing evidence that its beneficial effects are not solely due to its anticoagulatory effect [14]. For instance, APC has anti-apopotic effects [15,16], reduces nuclear translocation of nuclear factor κ-B [17], and can inhibit leukocyte adhesion by inhibiting leukocyte-induced arteriolar rolling [18]. However, it is unknown how rhAPC affects LPS-induced changes in the lung proteome.

Proteomics and difference gel electrophoresis (DIGE) permit simultaneous assessment of alterations in large numbers of proteins [19]. Proteomics has been used to describe LPS-induced changes in protein expression in animal models and human cell lines [20-25], and DIGE has recently been used to identify serial BALF proteome changes in ALI patients [26]. A limitation of previous studies of the human lung proteome has been the lack of an internal control which can limit the interpretation of findings for two reasons (1) the majority of BALF proteins are also plasma proteins [27] and (2) there is a large intersubject variability in expression of proteins [28]. In this study, we used a proteomic approach to identify changes in the lung proteome that result from intrapulmonary LPS with and without administration of rhAPC. The approach was uniquely powerful because each subject had saline instillation in a control lobe to eliminate inter-subject and temporal variability. This study provides insight not only into the alterations in pulmonary proteins induced by exposure to LPS, such as occurs with Gram negative pneumonia, but also identifies the changes in bronchoalveolar lavage protein profiles associated with administration of rhAPC.

## Methods

### Study subjects

All subjects were admitted to the General Clinical Research Center (GCRC) at the University of Colorado Hospital. Approval for this study was obtained from the Colorado Multiple Institutional Review Board, and informed consent was provided according to the Declaration of Helsinki. The subjects (N = 10) were randomly selected from a parent study designed to examine LPS-induced pulmonary neutrophilic inflammation with and without rhAPC (see [29] for details). The parent study subjects (N = 16) were non-smokers, ages 18–40 years, randomized to receive either rhAPC (drotrecogin alfa [activated]; 24 mcg/kg per hour) or normal saline (the solution for drotrecogin alfa [activated]) starting 2 hours before the initial bronchoscopy and continuing for 16 hours. The infusion of rhAPC or placebo was discontinued 2 hours prior to the second bronchoscopy to lessen the risk of hemorrhage resulting from anticoagulant properties of rhAPC. The volunteers were premedicated with one double-strength trimethoprim/sulfamethoxazole tablet 12 hours and 1 hour before bronchoscopy. Any subject with a history of sulfa allergy was given 2 doses of oral amoxicillin (500 mg), separated by 8 hours. Subject characteristics are listed in Table 1.

**Table 1:** Baseline characteristics of the subjects

	Control	rhAPC
N (f/m)	5 (4/1)	5 (2/3)
Age (years)	28 ± 8	27 ± 3
BALF protein (μg/mL) saline treated lobe	112 ± 66	80 ± 32
BALF protein (μg/mL) LPS treated lobe	456 ± 462	595 ± 235

Means and standard deviation (SD) of the groups are reported; there were no significant differences between groups

At the time of the first bronchoscopy, 10 mL saline was instilled into a lung subsegment (either the right middle lobe or lingula) followed by instillation of the test dose of reference endotoxin (4 ng/kg in 10 mL saline) into the contralateral lung. The subjects were randomized to left or right lungs for endotoxin or saline instillation. Following reconstitution of the endotoxin, a quantitative Limulus amebocyte lysate test was performed to verify the proper reconstitution and dosage of endotoxin. A second bronchoscopy was performed 16 hours after the initial bronchoscopy. At the time of the second bronchoscopy, both the endotoxin and placebo-instilled subsegments were lavaged with 150 mL normal saline.

### Processing of samples

The cell-free supernatants were frozen at -80°C. Total protein in BALF was determined by the bicinchoninic acid (BCA) assay and employed bovine albumin standards (Pierce, Rockford, IL). BALF aliquots of 3 ml each were dialyzed in 300 MWCO dialysis tubing (Pierce, Rockford, IL) against 4 L of 10 mM Tris-HCl pH 8.0 overnight at 4°C. The Tris-HCl was changed once after two hours. The dialyzed samples were freeze-dried and then resuspended in 7 M urea, 2 M thiourea, 1 M Tris-HCL pH 8.0, and 4% CHAPS. All reagents were supplied by Sigma Chemical Co. (St. Louis, MO) unless otherwise noted.

### Differential in-gel electrophoresis (DIGE)

For DIGE, 5 ug of protein was independently labeled with Cy3 and Cy5 dye as per the manufacturer's protocol (Amersham/GE). Following labeling both saline- and LPS-instilled BALF samples for each subject were combined and analyzed on the same gel. A dye swap was used for all samples to avoid labeling bias (i.e. for each subject a DIGE gel was run with control (saline) BALF sample labeled with Cy3 labeled and LPS sample labeled with Cy5 and a second DIGE gel was run with control sample labeled with Cy5 and a LPS sample labeled with Cy3). Dye labeling occurred in the dark at 37°C and was stopped with 7 M urea, 2 M thiourea, 4% CHAPS, DTT 130 mM, and 2% biolytes. For protein spot identification, 10 μg of each sample was pooled and then 500 μg total used for subsequent steps (preparative gel).

The combined samples were applied to 24 cm immobilized pH gradient (IPG) strips (BioRad) with a pH 3–10 linear gradient. Rehydration took place at 50 V for 17 h and at 20°C in a Protean isoelectric focus (IEF) Cell (BioRad). First dimension isoelectric focusing was carried out at 20°C in a Protean IEF Cell using the following protocol: 250 V for 15 min; 10,000 V for 3 hours; then overnight at 10,000 V for 60,000 volt-hours followed by a 500 V hold. After isoelectric focusing, strips were equilibrated by agitating for 10 minutes in 50 mM Tris-HCl, pH 8.8, 6 M urea, 30% (v/v) glycerol, 2% (w/v) sodium dodecyl sulfate (SDS), 650 mM DTT and then agitating for10 minutes in 50 mM Tris-HCl, pH 8.8, 6 M urea, 30% (v/v) glycerol, 2% (w/v) SDS, 1.27 M iodoacetamide. The strips were next loaded onto a 24 × 24 cm 12% polyacrylamide gel using low fluorescence glass plates and subjected to an electric field in a Protean Plus Dodeca cell (BioRad; 4°C at a constant 20 mA per gel, 18 hours in a running buffer containing 25 mM Tris, 192 mM glycine, and 0.1% (w/v) SDS). The preparative gel was stained with Sypro Ruby (BioRad) as previously described [10]. After electrophoresis, proteins were digitally imaged using a Typhoon Variable Mode Imager (GE Healthcare).

### Image analysis

Images were analyzed using DeCyder 5.0 (GE Healthcare). Spot volumes (Area × Density) were calculated for each spot and averaged across the gel. The volume ratios for the control and LPS BALF were calculated for each spot. Spots that increased or decreased at least two-fold in both dye swap gels in at least two subjects were selected for identification.

### Protein identification

Protein identifications were made using a pooled preparative gel and stained with Sypro Ruby (Biorad). Spots of interest were excised using an Ettan Spot Picker (GE Healthcare) and digested overnight with trypsin using an automated basic protocol and Ettan Digester (GE Healthcare). Extracted peptides were subsequently dried to reduce volume and remove organic solvent. Peptides were chromatographically resolved on-line using a C18 column and analyzed using a 6340 LCMS ion trap mass spectrometer (Agilent Technologies, Palo Alto, CA) in the National Jewish Health Proteomics Facility. The mass spectrometry system includes a high performance liquid chromatography (HPLC) chip interface (Agilent Technologies). Raw data was extracted and searched using the Spectrum Mill search engine (Rev A.03.03.038 SR1, Agilent Technologies, Palo Alto, CA). "Peak picking" is performed within SpectrumMill with the following parameters: signal-to-noise is set at 25:1, a maximum charge state of 4 is allowed (z = 4), and the program is directed to attempt to "find" a precursor charge state. During searching the following parameters were applied: IPI human database (IPI Human release 3.28, 16-APR-2007), carbamidomethylation as a fixed modification, trypsin, maximum of 1 missed cleavage, precursor mass tolerance +/- 1.7, product mass tolerance +/- 0.7, and maximum ambiguous precursor charge = 3. Data were evaluated and protein identifications were considered significant if the following confidence thresholds were met: minimum of 2 peptides per protein, protein score > 20, individual peptide scores of at least 7, and Scored Peak Intensity (SPI) of at least 70%. The SPI provides an indication of the percent of the total ion intensity that matches the peptide's MS/MS spectrum. A reverse (random) database search was simultaneously performed and manual inspection of spectra was used to validate the match of the spectrum to the predicted peptide fragmentation pattern, hence increasing confidence in the identification. Standards are run at the beginning of each day and at the end of a set of analyses for quality control purposes.

### Statistical analysis

Protein spots for the BALF proteome from the LPS-instilled lobe were matched to the BALF proteome of the saline-instilled lobe. JMP version 7.0 for Macintosh (SAS Institute, Cary, NC) was used to compute medians, means and standard deviations for relative increases or decreases in protein abundance. Clusters of protein spots with identical identifications were grouped together for analysis since they all had nearly identical relative changes in abundance. A non-parametric test (Wilcoxon Matched-Pairs Rank-Sum Test) was used to determine if relative changes were significantly different at a predetermined significance level of P < 0.05.

## Results

**Figure 1:**
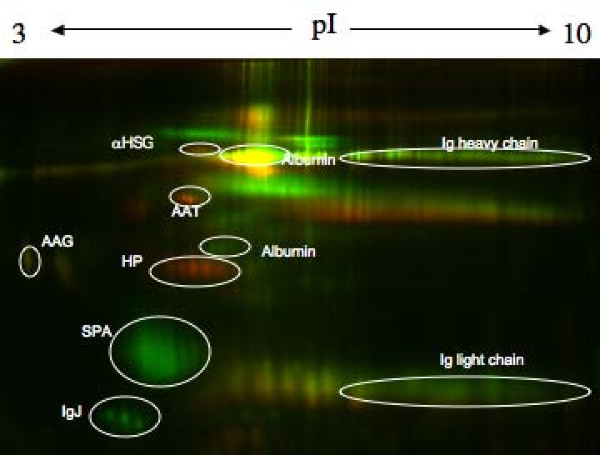
**A pseudocolor difference gel electrophoresis (DIGE) image from a representative subject (subject 19)**. Proteins from the saline treated lung lobe were conjugated to Cy3 (shown in green) and protein from the LPS treated lobe was conjugated to Cy5 (shown in red). Equal amounts of protein were loaded onto the gel from each condition. Green spots represent proteins that were relatively higher in the saline treated lobe; red spots indicate proteins that were relatively higher in LPS treated lobe; yellow spots represent proteins that were expressed at relatively similar levels. Each of the circled spots was identified by mass spectrometry and labeled according to identification as follows: haptoglobin (HP), surfactant protein A (SPA): immunoglobulin J chain (IgJ), α_1_-antitrypsin (AAT), fibrinogen-γ (Fγ), α2-HS-glycoprotein (αHSG), immunoglobulin G (IgG).

**Figure 2:**
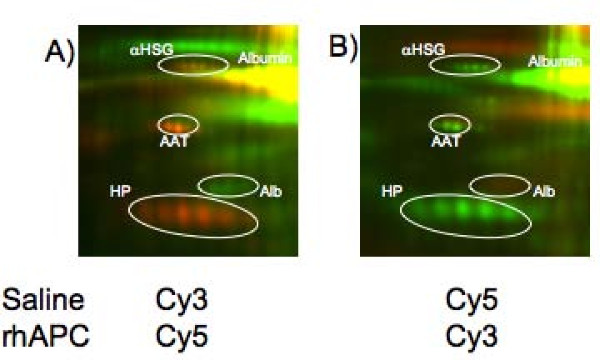
**Representative dye swap experiment shows that changes in relative protein expression are not due to differential labeling for Cy3 or C5**. (A) Proteins from the saline treated lobe were conjugated to Cy3 (shown in green) and protein from the LPS treated lobe were conjugated to Cy5 (shown in red). (B) Proteins from the saline treated lobe were conjugated to Cy5 (shown in red) and protein from the LPS treated lobe were conjugated to Cy3 (shown in green). Relative changes in protein expression were of identical direction and similar magnitude regardless of which dye was used to label proteins.

BALF protein concentrations were significantly higher in lobes exposed to LPS (526 ± 110 μg/ml) compared to those that received saline (90 ± 16 μg/ml; P < 0.001); however, there were no significant differences in BALF protein concentration in subjects who received rhAPC versus those who received saline. An average of 255 spots (range 204–307) were identified and used for analysis for each gel. A representative gel is shown in Figure 1. There were no significant differences in the number of spots automatically detected in subjects that received saline versus rhAPC. Relative changes in protein expression were independent of dye labeling for nearly all proteins (Figure 2). Protein spots that had relative increases or decreases of at least 2-fold at P < 0.05 in at least six of the subjects were picked for identification (Table 2). Most proteins were associated with a train of spots that yielded identical identifications by mass spectrometry. For example, IgJ protein was represented by three spots. In all cases, these protein spots behaved as a group: e.g. when one protein spot increased two-fold all other spots in the group increased approximately two-fold. Both median and mean intensity changes were reported for the group as a whole (Table 3) and also broken down by whether they received saline or rhAPC (Table 4). We have previously identified other proteins such as transferrin, clusterin, serum amyloid protein, hemoglobin α-2, and α-2 HS-glycoprotein that were elevated in patients with acute lung injury [10]; however, these proteins were not differentially abundant in this study. The following describes the proteins that we identified as being differentially abundant in BALF with LPS-instillation:

**Table 2:** Characteristics of proteins that significantly changed in the lobe treated with saline vs LPS

							Observed	
Protein	Abbreviation	Accession #	Score	Peptides	Coverage	Identification Method	Mass (kD)	pI
Alpha 2-HS glycoprotein	αHSG	IPI00020091	52	4	19%	LCMS	68	4.8
α1-antitrypsin	AAT	IPI00553177	62	5	14%	LCMS	43	5.7
Albumin	Alb	IPI00022434	108	8	11%	LCMS	42	5.7
Fibrinogen γ	Fg	IPI00021891	84	7	37%	MALDI	60	5.8
Haptoglobin	HP	IPI00641737	41	3	6%	LCMS	44	5.5
Immunoglobulin	IgG-l	IPI00641082	50	5	20%	LCMS	26	7.5–8.1
Immunoglobulin J chain	IgJ	IPI00178926	35	3	16%	LCMS	29	4.6
Surfactant Protein A	SPA	IPI00012889	46	2	11%	LCMS	35	5.0

**Table 3:** Magnitude of protein changes in saline versus LPS treated lobe

	Median (Mean) fold change in LPS treated lobe compared to saline treated lobe	
Protein	decrease	increase
Alpha 2-HS glycoprotein	2.38 (3.3)	
α1-antitrypsin	2.4 (3.0)	
Albumin	2.4 (2.8)	
Fibrinogen γ	1.6 (1.6)	
Haptoglobin		2.58 (5.76)
Immunoglobulin	2.0 (2.2)	
Immunoglobulin J chain	3.79 (9.6)	
Surfactant Protein A	2.0 (14.3)	

Note that each patient was his/her own control and that changes are expressed as relative abundance of the protein in the proteome of the LPS treated lobe compared to relative abundance of the protein in the proteome of the saline treated lobe

**Table 4:** Median (mean) relative abundance of BALF proteins in saline versus LPS treated lobe in control and rhAPC treated subjects

Protein	Control	rhAPC	P
Alpha 2-HS glycoprotein	-1.36 (-2.2)	-2.74 (-4.3)	< 0.05
α1-antitrypsin	-1.35 (-1.2)	-2.55 (-3.7)	< 0.05
Albumin	-1.3 (-2.2)	-3.6 (-4.1)	NS
Fibrinogen γ	-1.3 (-1.1)	-1.87 (-2.2)	< 0.01
Haptoglobin	3.18 (8.2)	2.06 (3.3)	NS
Immunoglobulin J chain	-2.55 (-1.7)	-14.06 (-17.0)	< 0.001
Surfactant Protein A	-1.94 (-6.0)	-20.78 (-22.5)	NS

Negative fold changes represent decreases while positive fold changes represent increases; P values calculated using Wilcoxan-Rank-Sum

### Haptoglobin (HP)

We detected 5 protein spots for haptoglobin in all 10 subjects. In 9 of 10 subjects there was an increase in haptoglobin protein spot intensity in the LPS lobe, with a median increase of 3.18-fold (95% range 1.3–25.9-fold) in the subjects who received saline versus a 2.07-fold increase (95% range 0.25–17.9-fold) in subjects that received rhAPC (P = 0.053). Representative database results for HP were as follows: Accession number: IPI00641737, Score: 41.37, 3 peptides (scores 20,12, and 9, and SPIs 98.3, 86.6, and 96.5 respectively), and 6% total coverage.

### Surfactant Protein A (SP-A)

There were 5 protein spots detected for SPA in 9 of 10 subjects. One subject in the control group did not have SP-A detectable in either the control or LPS instilled lobe BALF. In 7 of 9 of the subjects there was a decrease in SP-A protein spot intensity in the LPS lobe with a median decrease of 1.94-fold (95% range 0.8–44-fold decrease) in the subjects who received saline versus a median 20.78-fold decrease (95% range 0.3–78-fold decrease) in subjects that received rhAPC (P = NS). Database results for SP-A were as follows: Accession number: IPI00012889, Score: 45.53, 2 peptides with multiple spectra (scores 24 and 22, and SPIs 96% and 97.5% respectively), and 11% total coverage.

### Immunoglobulin J chain (IgJ)

There were 3 protein spots detected for IgJ in 8 of 10 subjects. Two subjects in the control group did not have IgJ detectable in either the control or LPS instilled lobe BALF. All 8 of the remaining subjects had a decrease in IgJ protein spot intensity after LPS with a median decrease of 2.55-fold (95% range 0.3–3.6 fold decrease) in the subjects who received saline versus a median 14.06-fold decrease (95% range 3.8–48.5-fold decrease) in subjects that received rhAPC (P < 0.001). Database results for IgJ were as follows: Accession number: IPI00178926, Score: 34.68, 3 peptides with multiple spectra (scores 17, 9.45 and 7.9, and SPIs 96%, 97%, and 95% respectively), and 16% total coverage.

### Albumin variant

There were 3 protein spots for an albumin fragment detected in 8 of 10 subjects. Although the calculated mass of this protein was 71.6 kD and pI 6.33, the observed mass was 42 kD and the pI 5.3 suggesting that there was a post-translational modification. Two subjects in the group that received saline did not have the albumin fragment detectable in either the control or LPS instilled lobe BALF. In 7 of 8 of the subjects there was a 2-fold decrease in albumin fragment protein spot intensity after LPS with a median decrease of 1.3-fold in the subjects who received saline versus a 3.6-fold decrease in subjects that received rhAPC (P = 0.05).

### α_1_-antitrypsin (AAT)

There were 3 protein spots for AAT detected in all 10 subjects. In 8 of the subjects there was a decrease in AAT protein spot intensity in the LPS instilled lobe. Two of the subjects treated with rhAPC had a less than 2-fold decrease in AAT. The median decrease in AAT was 1.35-fold (95% range 0.4–3.4-fold decrease) in the subjects who received saline versus a 2.55-fold (95% range 1.9–12.8-fold decrease) in subjects that received rhAPC (P < 0.05). Database results for ATT were as follows: Accession number: IPI00553177, Score: 62.23, 5 peptides from multiple spectra (scores 18, 14, 11, 10, and 10 and SPIs 86%, 79%, 74%, 87%, and 75% respectively), and 14% total coverage.

### Fibrinogen-γ (Fγ)

There was 1 protein spot for Fg detected in all 10 subjects. In 9 of the subjects there was a decrease in Fγ protein spot intensity after LPS, with a median decrease of 1.3-fold (95% range 0.7–7.0 fold decrease) in the subjects who received saline versus a 1.87-fold decrease (95% range .1.2–4.7 fold decrease) in subjects that received rhAPC (P < 0.01). Fγ was identified as previously described [10].

### α2-HS-glycoprotein (αHSG)

There were 3 protein spots detected for αHSG in all 10 subjects. All of the subjects had a decrease in αHSG protein spot intensity in the LPS-instilled lobe with a median decrease of 1.36-fold (95% range 0.7–7.0-fold decrease) in the subjects who received saline versus a 2.74-fold decrease (95% range 1.2–15.1-fold decrease), in subjects that received rhAPC (P < 0.05). Database results for aHSG were as follows: Accession number: IPI00020091, Score: 52.34, 4 peptides from multiple spectra (scores 19, 14, 11, and 8 and SPIs 96%, 96%, 81%, and 71% respectively), and 19% total coverage.

### Immunoglobulin G (IgG)

There were more than 10 protein spots detected for IgG in all 10 subjects. In 4 of 5 of the control subjects there was no change in IgG light chain (IgG-l) protein spot intensity after LPS with a median increase of 1.0-fold. All 5 subjects given rhAPC had a greater than 2-fold decrease in IgG-l protein spot intensity of 3.1-fold (P = 0.003 compared to control group). IgG heavy chain (IgG-κ) was detected in all 10 of 10 subjects; however, for unclear reasons, labeling of the proteins with Cy3 was 10-fold more efficient than with Cy5. Thus, the dye swap revealed that DIGE is unreliable for detecting differences in this protein.

### Other proteins

There is a substantial amount of albumin in BALF. However, there were no relative changes in albumin abundance after LPS other than the fragment discussed above. Unlike one of our previous studies of ALI subjects [10], there were no significant differences in expression of α_1_-acid glycoprotein.

## Discussion

This study demonstrates that instillation of intrapulmonary LPS results in significant changes in the relative abundance of selected proteins. The approach we used is unique because to our knowledge it is the only BALF proteomics study in which the research subject is his/her own control, thereby greatly decreasing the "noise" of biologic variation. The observed changes in relative abundance are unlikely to be due to external factors for the following reasons: (1) scanning protocols were optimized to avoid fluorescent saturation during image analysis (saturation leads to underreporting of differences of abundant proteins); (2) we performed dye-swap experiments for all subjects to eliminate the potential bias of differential labeling with Cy3 versus Cy5 dye; (3) the direction of change in relative abundance and the magnitude of change were similar for all studied proteins. Furthermore, intersubject and temporal variability were eliminated by using a saline instilled lobe as an internal control for each subject. It is possible but unlikely that the observed changes in relative abundance were due to technique (the bronchoscopist and proteomics technicians were blinded to group), random chance (some of the findings have been reported in clinical studies of LPS and ALI), or that saline instillation induced changes that masked LPS-induced changes. Thus, the most likely explanation for the observed changes is true biologic changes in the pulmonary proteome in response to LPS. Although we confirmed that the largest biologic factor that affects the absolute content of protein in the BALF is a breakdown in the alveolar-capillary barrier [30], the *relative abundance *of the majority of high molecular weight proteins in the lung proteome did not change suggesting that the observed relative changes in abundance may be due to factors other than breakdown in the alveolar-capillary barrier.

Although this study was conducted under uniform experimental conditions and used internal controls, our findings can be contrasted with other uncontrolled lung proteomic studies of health and sick individuals in a clinical setting. For instance, Wattiez et al [31] studied the BALF proteome from 5 healthy subjects, 3 patients with idiopathic pulmonary fibrosis (IPF), and 2 patients with hypersensitivity pneumonitis. Similar to our findings, these investigators were able to detect relative decreases in the intensity of SP-A in patients with IPF and HP, but found relative increases in the intensity of proteins, such as transferrin, transthyretin, α_1_-AT, and immunoglobulin. The IPF and hypersensitivity pneumonitis patients also demonstrated numerous spots corresponding to the N-terminal sequences of haptoglobin. In another study of 8 healthy children and 17 children with cystic fibrosis, there was increased intensity of α_1_-AT and lower molecular weight isoforms of SP-A [32]. In an analysis of BALF collected from seven healthy children undergoing diagnostic operations and 10 children with malignancies, fever, and chest infiltrates, the sick children were noted to have large increases in intensities of α_1_-AT and hemoglobin, decreases in transthyretin, but no changes in SP-A, transferrin, or immunoglobulins [33]. We have also previously reported that patients with acute lung injury (ALI) have changes in SP-A and acute phase proteins, such as haptoglobin and α_1_-AT [10]. Relative decreases in BALF SP-A in ALI patients have also been reported in other studies and have prognostic value [34,35]. In all of these studies it was unclear whether these changes were biased because there were no internal controls of the lung proteome in an unperturbed state. Our investigation used each subject as his/her own control, thus these problems were eliminated. However, our results did show similarities in some of the lung proteome changes (e.g. LPS instillation was associated with relative decreases in SP-A and increases in haptoglobin).

One of the hallmarks of nearly all of these forms of lung injury is loss of size selectivity of the alveolar-capillary barrier [36] which causes high MW proteins, including albumin, transferrin, and immunoglobulin, to leak from plasma resulting in an increased concentration of protein in the airspaces [7]. The 6-fold increase in total protein concentration that we observed in the LPS instilled lobe is consistent with these findings; however, a change in the alveolar epithelial barrier cannot explain relative changes in protein abundance for selected proteins because the majority of larger proteins (e.g. most albumin variants, IgG) did not show changes in relative abundance in the LPS-instilled lobe. The most likely explanation for the lack of change in relative abundance of the majority of these proteins is that the DIGE approach we used only measures larger (>10 kD) proteins, thus low molecular weight proteins did not contribute to the analysis and loss of size selectivity of the alveolar-epithelial barrier became less relevant. Other limitations to the DIGE procedure include a bias towards detecting high and medium abundant proteins and an inability to quantitate proteins that are insoluble or do not stain (see review [19]). Since no experimental technique can detect or quantitate all proteins at all concentrations, we acknowledge that there may be changes in the proteome that could have been missed by this technique.

An alternative to the loss of alveolar-capillary barrier integrity explanation is the hypothesis that LPS directly alters lung metabolism of specific proteins. Supporting this hypothesis are animal models demonstrating that intrapulmonary LPS induces lung mRNA expression of serum amyloid protein, β-2 glycoprotein-1, α-2 glycoprotein 1, and α-2 HS-glycoprotein; however, this response is complicated by systemic mediators (e.g. IL-6) that may also influence the expression of these proteins in the liver [37]. Indeed an increase in systemic acute phase proteins is one of the hallmark responses to inflammation [38]. In our experimental model, the use of an internal control (saline instilled lobe) should correct for these systemic responses.

One example of a "plasma" protein whose metabolism can be altered in the lung itself is haptoglobin. Haptoglobin is well known to complex with hemoglobin and is metabolized in the hepatic reticuloendothelial system; however, haptoglobin also has antioxidant and antimicrobial activity and plays a role in host defense responses to infection and inflammation, acting as a natural antagonist for receptor-ligand activation of the immune system [39]. The lung epithelium is a major source of extra hepatic haptoglobin gene expression and can be induced by LPS [40]. Although a proteomics approach cannot differentiate the sites of origins of proteins, we observed a relative increase in haptoglobin in the LPS-instilled lobe suggesting that the source might be from pulmonary origin. Surprisingly, there have been few studies of the role of haptoglobin in the lung [41] suggesting that the role of this protein in the lung injury response needs further investigation.

Unlike haptoglobin, SP-A has a well-described pulmonary origin from type II epithelial cells. Our observations of a relative decrease in SP-A are consistent with many findings in lung injury studies in humans. For instance, pulmonary SP-A has been previously shown to be depressed in patients with acute lung injury [10,34] and plasma SP-A has been shown to have prognostic value [35]; however, two studies of rats reported an increase in SP-A after LPS [42,43], and a study of human edema fluid SP-A in ALI patients did not find that SP-A had prognostic implications [35]. Furthermore, impairment of SP-A function appears to increase over time from onset of ALI [44]. These studies highlight that it is important to consider the animal model (e.g. mouse/rat/human), sampling technique (BALF/edema fluid), and time course when interpreting results. Despite this, the majority of evidence suggests that SP-A is decreased in ALI. The mechanisms by which LPS down regulates SPA is unknown, but may be due to binding and clumping to the lipid portion of LPS [45] or enhanced clearance from excess nitration [46].

One systemic protein that may have immune implications in the lung is IgJ. IgJ is a linker protein for IgM and IgA, but not IgG. It is expressed in lung; however, most expression is in lymphocytes (Novartis GNF SymAtlas database). We observed changes in relative abundance of IgJ protein suggesting that there may be a decrease in relative abundance of IgA or IgM immediately after LPS exposure. Although not well studied in the lung, one explanation could be due to binding of these proteins to known receptors on airway epithelial cells [47].

Fibrin deposits are a major hallmark of lung injury and local fibrin consumption through activation of coagulation occurs in lung injury. We observed a relative decrease in fibrinogen-γ in the LPS-instilled lobes compared to the saline-instilled lobes. Treatment with rhAPC was associated with slightly larger relative decreases in fibrinogen-γ in the LPS-instilled lobes, possibly due to rhAPC's PAI-1 inhibiting effects and restoration of the deficient clearing of fibrin. In humans there is an inverse relationship between fibrinogen and APC [48], thus rhAPC infusions may lead to a relative decrease in fibrinogen concentrations with the lung. In heatstroke models, there have been conflicting results with a rat model showing improved fibrinolysis [49], but no change in fibrinolysis in a baboon heatstroke model [50]. Furthermore, in septic humans there were no significant changes in fibrinogen with rhAPC [51]. The role of rhAPC with other acute phase reactants such as antitrypsin is unclear since AAT inhibits APC activity [52], although this is not through the 37 loop of APC [53].

The otherwise modest changes that we observed in the lung proteome of rhAPC patients is consistent with other studies that have found minimal differences in volunteers given intravenous LPS and rhAPC [11] as well as clinical studies in patients with severe sepsis but low risk of death [54]. The lack of reduction in total BALF protein increase in rhAPC-pretreated patients is also consistent with some [55] but not all [56] mouse models of LPS induced lung injury. In clinical studies, the beneficial role of rhAPC has been most marked in patients with severe sepsis [57]; however, the volunteers in this study were unlikely to have had the severe systemic changes that occurred in these patients. Furthermore, rhAPC is not an effective treatment for acute lung injury [58]. The experimental model in this study is one of acute lung (more similar to pneumonia). Thus, modest changes that we observed in the subjects who received rhAPC versus the saline should not be generalizable to its role of rhAPC in severe sepsis.

## Conclusion

Instillation of LPS into human lung results in stereotypical patterns of increased or decreased relative abundance of selected proteins; however, the magnitude of these changes varies among individuals. These observations are particularly relevant because each subject served as his/her control, thus eliminating intersubject variability. The relative changes in the selected proteins suggest that LPS induces specific metabolic changes in the lung besides just a disruption in the alveolar epithelial barrier. Lung specific alterations in some of these proteins (e.g. SP-A and haptoglobin) could serve as targets for novel therapies in future research; however, rhAPC treatment was associated with only modest changes in the LPS response, consistent with its lack of clinical benefit in ALI or modest sepsis.

## Abbreviations

AAT: α_1_-antitrypsin; ALI: acute lung injury; BALF: bronchoalveolar lavage fluid; DIGE: difference gel electrophoresis; Fγ: fibrinogen gamma; GNS: Gram negative sepsis; HP: haptoglobin; LPS: lipopolysaccharide; rhAPC: Recombinant human activated protein C; SP-A: surfactant protein A.

## Competing interests

This work was supported by a research grant from Lilly to RB. There are no competing interests for NR, RR, or EA.

## Authors' contributions

RB conducted the 2 DIGE, quantitation, protein isolation and statistics. NR and RR performed the protein identification. EA conducted the parent study and provided the samples and clinical information. All authors contributed to the drafting and revising of the manuscript.

## Pre-publication history

The pre-publication history for this paper can be accessed here:


